# Comparing Brain Networks of Different Size and Connectivity Density Using Graph Theory

**DOI:** 10.1371/journal.pone.0013701

**Published:** 2010-10-28

**Authors:** Bernadette C. M. van Wijk, Cornelis J. Stam, Andreas Daffertshofer

**Affiliations:** 1 Research Institute MOVE, VU University Amsterdam, Amsterdam, The Netherlands; 2 Department of Clinical Neurophysiology, VU University Medical Center, Amsterdam, The Netherlands; Indiana University, United States of America

## Abstract

Graph theory is a valuable framework to study the organization of functional and anatomical connections in the brain. Its use for comparing network topologies, however, is not without difficulties. Graph measures may be influenced by the number of nodes (*N*) and the average degree (*k*) of the network. The explicit form of that influence depends on the type of network topology, which is usually unknown for experimental data. Direct comparisons of graph measures between empirical networks with different *N* and/or *k* can therefore yield spurious results. We list benefits and pitfalls of various approaches that intend to overcome these difficulties. We discuss the initial graph definition of unweighted graphs via fixed thresholds, average degrees or edge densities, and the use of weighted graphs. For instance, choosing a threshold to fix *N* and *k* does eliminate size and density effects but may lead to modifications of the network by enforcing (ignoring) non-significant (significant) connections. Opposed to fixing *N* and *k*, graph measures are often normalized via random surrogates but, in fact, this may even increase the sensitivity to differences in *N* and *k* for the commonly used clustering coefficient and small-world index. To avoid such a bias we tried to estimate the *N*,*k*-dependence for empirical networks, which can serve to correct for size effects, if successful. We also add a number of methods used in social sciences that build on statistics of local network structures including exponential random graph models and motif counting. We show that none of the here-investigated methods allows for a reliable and fully unbiased comparison, but some perform better than others.

## Introduction

First reports of small-world network structures [1] and scale-free networks based on preferential attachment [2] have inspired many researchers investigating the anatomical and functional organization of the nervous system. The introduction of graph theory to neuroscience opened a new window into the study of complex neural network organizations. In the past decade, small-world networks have been found for the anatomical connections in *C. elegans* [1], cat cortex, and macaque (visual) cortex [3]. In humans, anatomical connectivity *in vivo* has been successfully assessed via cross-correlation analysis of cortical thickness (grey matter) in structural MRI [4], [5], revealing topological differences between healthy controls and patients with Alzheimer's disease [6] and schizophrenia [7]. Recent advances in tractography allowed for more direct studies of anatomical network structure (white matter) based on diffusion tensor imaging and diffusion spectrum imaging [8]–[10]. Small-world networks are known for their efficiency in that they enable a rapid integration of information from local, specialized brain areas even when they are distant [3]. The significance of efficient brain network topology is emphasized by reports of correlations between corresponding graph measures and intelligence [11]–[13].

In contrast to anatomical connections, functional connections may evolve on a much quicker time scale and can reveal information on network organization underlying specific brain functions. So far, however, small-world organizations in human functional networks have primarily been studied in resting state, either using fMRI [14]–[17] or M/EEG [18]–[21]. Abnormalities in these resting state networks appear to relate to neurological and psychiatric diseases: graph measures differ between patients and healthy controls in Alzheimer's disease [22]–[25], schizophrenia [26], [27], ADHD in children [28], and brain tumors [29], [30]. Also, changes in small-worldness have been found at slow and fast time scales alike, e.g., as effect of aging [31]–[33], in different sleep stages [34], [35], and during epileptic seizures [36]–[38]. To explicitly study their task-dependence, changes in graph measures have been studied when subjects performed foot movements [39] and finger tapping [19].

**Figure 1:**
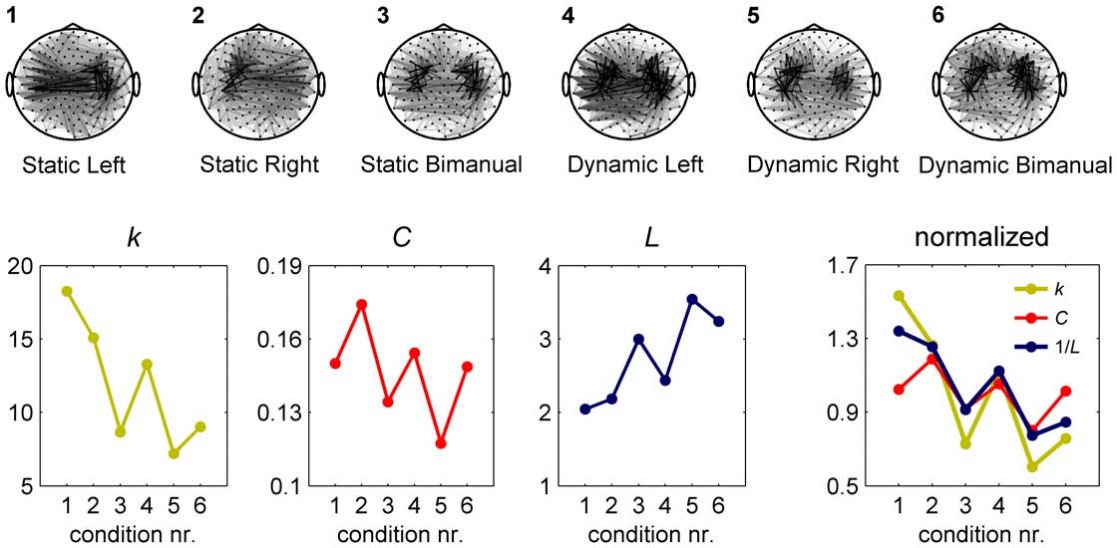
Differences in average degree between empirical networks may influence graph measures. The data here are taken from an MEG experiment we conducted in which participants performed a precision grip force in six experimental conditions that differed in the force pattern exerted (static or dynamic) and hand used (left, right, or bimanual). The topographies show the relative phase uniformity (15–30Hz) between all MEG sensor combinations that is increased compared to resting state. The gray scale indicates the connectivity strength, with stronger connections in darker colors. Results are averaged over 3×40s steady state force production and 20 participants. Lower panels: although the clustering coefficient (*C*) and path length (*L*) show clear differences between conditions, these effects co-vary with differences in average degree (*k*). This can be seen more clearly in the right plot where values are divided by the average for each measure and *L* is inverted. It is difficult to disentangle true experimental effects from those introduced by differences in *k* because the exact dependence of *C* and *L* on *k* is unknown.

While all these results are interesting in their own right and are very promising for up-coming research throughout neuroscience, the general methodology of comparing network structures or network topologies of different systems appears a challenge, certainly across studies but also within a single experimental design. As will be explained in detail below, topologies can be estimated using various graph measures. Central to our studies is the fact that most of these measures depend on network size. If network size is altered, i.e. if numbers of nodes (*N*) and/or connections (average degree *k*) are changed, then the graph measures will differ even if the network topology remains identical (Figure S1). In addition, such size effects (*N*,*k*-dependence) differ for different topologies. That is, to ‘correct’ graph measures for size effects, the underlying topology needs to be known, which is typically not the case for empirical data. However, if size effects are hard to tackle, it is certainly difficult to discriminate between the real effects of experimental manipulations and the effects of simply changing network size and/or density between conditions, as illustrated in Figure 1. When reviewing the literature it appears that these size and degree effects are often overlooked or at least underestimated.

How can networks of different size and connectivity density be accurately compared? Unfortunately, we cannot give a definite answer to that crucial question since, yet, an approved, unbiased method for empirical data does not exist. That is, one runs the risk of obtaining a bias in the comparison between network topologies and, hence, misinterpretation of results. The purpose of this paper is hence to review advantages and disadvantages of methods commonly used when estimating the topology of neural connectivity and to supplement this by several alternative approaches to compare networks, including ones that are used in social network research.

## Analysis

### Graph analysis

We first provide a brief description of fundamental steps and notations in graph analysis that will serve as glossary for the following sections. Networks can, in general, be represented as graphs that consist of *nodes* and their connections, here referred to as *edges*. From these representations, a variety of graph *measures* can be calculated that are informative about the network *topology*. We used several standard measures to describe the network's topology as they were also summarized by, e.g., Watts and Strogatz [1]: the *average degree* (*k*), which denotes the average number of edges per node; the *degree distribution*, which indicates the distribution of the network's nodal degree values; the *characteristic path length* (*L*), which is the average number of edges in the shortest paths between every pair of nodes in the network; and, the *average clustering coefficient* (*C*) that represents the probability that neighbors of a node are also connected. *C* indicates the occurrence of clusters or cliques in the network. Exact definitions of these measures can be found in Text S1, where we also added several other, less common measures (for a recent overview of graph measures used in neuroscience see, e.g., [40]).

We note that by ‘topology’ we mean the layout of a realization of a particular generating model, e.g., the Watts-Strogatz's rewiring model for small-world networks, with fixed parameters other than *N* and *k*. Topologies are hence equivalent when derived from the same baseline model irrespective of network size and density. Since the only parameters of Erdös-Rényi random networks and lattices are *N* and *k*, this means that we consider all Erdös-Rényi networks to have the same topology (all random) and all lattices to have the same topology (all regular). By contrast, two Watts-Strogatz small-world networks with a different rewiring probability have distinct topologies.

#### *N,k*-dependencies in known network topologies

In order to illustrate the size- and degree-dependence of graph measures we list (approximations of) *L* and *C* for several canonical topologies. Although these theoretical networks are unlikely to be found in empirical data we do capitalize on their mathematical forms because they can provide a good feel about possible changes in the one or the other measure. Expressions depend either on *k* or *N*, or both and are specific for a particular network type. For instance, the path length *L* depends linearly on *N* and reciprocally on *k* for lattices, depends logarithmically on *N* and *k* for Erdös-Rényi random networks, and has a double-logarithmic *N*-dependence for Barabási-Albert scale-free networks; see Table 1 for more details.

**Table 1:** Analytical expressions for graph measures of theoretical networks.

Network type	Path length (*L*)	Clustering coefficient (*C*)
Ring lattice	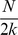	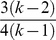
Random networkErdös-Rényi	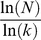	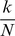
Small-world network with rewiring probability *p*Watts & Strogatz	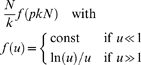	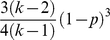
Scale-free networkBarabási-Albert	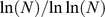	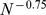

Expressions adapted from [80].

### Defining graphs from data

Most studies consider undirected, unweighted (i.e. binary) networks. Voxels, specific regions of interest, or electrodes are taken as nodes and their number is usually fixed throughout an experimental design. Subsequently, connectivity values are estimated between all possible pairs of nodes based on, e.g., correlation or coherence between the corresponding time series. A threshold is applied to convert these values into edges, i.e. if above threshold then an edge exists, otherwise it is absent. This results in a binary *adjacency matrix* from which the aforementioned graph measures can be calculated. Reason to convert connectivity to binary values in the first place is to enhance the contrast between relevant and irrelevant values, since the first are only based on the most important values, at least if the threshold is chosen properly.

The number of nodes in the network, i.e. the size of the adjacency matrix, depends on the number of recording sites and/or parcellation scheme [41]–[43]. For EEG and MEG this will typically be in the range from 16 to 150 nodes. Likewise, for MRI based methods relatively small scale parcellation schemes are usually adapted like AAL (Automated Anatomical Labeling, containing 90 nodes) or ANIMAL (Automatic Nonlinear Imaging Matching and Anatomical Labeling, containing 70 nodes), although voxel-based approaches have also been performed. As will be shown below, it is often for these small numbers of nodes when effects of different network sizes cannot be ignored. Although usually the network size is fixed within a single experiment, it does complicate the comparison between studies using different data sources. The average degree is determined by the way of thresholding connectivity values. In the following paragraphs we sketch how different approaches for constructing the adjacency matrix may influence results.

#### Fixed threshold: *k*-dependence

In general, the choice of threshold should depend on the research question and falls in the regime of educated guesses, especially when simply fixing to a certain value. Three criteria are typically adopted: (1) one uses a 5% significance level as a threshold in order to omit connectivity values that can readily be expected by chance (e.g., [5], [14], [17], [27]); (2) one selects just an arbitrary value as threshold, with which one roughly obtains a certain desired average degree of the network (e.g., [12], [32], [36]); or, (3) one defines the threshold as large as possible while guaranteeing that all nodes are connected or a so-called giant component exists [19]. However, connectivity values often vary between subjects and conditions, which may yield a clear difference in the total (summed) connectivity. This can result in a difference in average degree *k* when using the same fixed threshold for all networks under study. Most studies reflect awareness to this problem as they include multiple thresholds, determine the most ‘appropriate’ threshold value by different means, or even show how graph measures may fluctuate as a function of threshold. Although results could be preserved over a broad range of thresholds, the problem of an accurate comparison remains as differences in *k* between networks are also preserved over the same range of thresholds.

#### Fixed average degree: network structures may change

**Figure 2:**
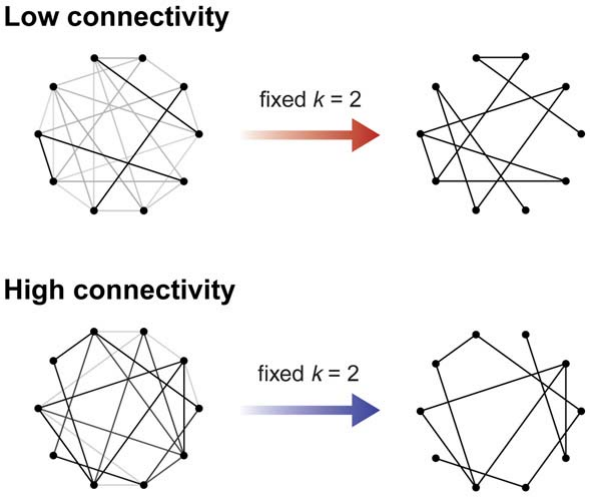
Imposing a fixed average degree can modify network structure. Applying a variable threshold to obtain a fixed average degree (*k*) may have consequences for the resulting network structure. A relatively large average degree for a network with low overall connectivity will convert non-significant values into edges. By contrast, a relatively small *k* for a network with high overall connectivity will omit a number of significant connections. Connectivity strength is indicated using a gray scale with black indicating strong (significant) connections.

To omit all *k*-dependencies one may adjust the threshold for each individual network so that *k* is fixed over all recordings and conditions (e.g., [6], [25], [29], [34], [35]). However, as said, the overall connectivity in empirical networks can vary profoundly, rendering the fixed average degree *k* generally problematic. A fixed *k* may be relatively large for a network with low average connectivity and, by the same token, relatively small for a network with high average connectivity. Put differently, for a network with low average connectivity there will be fewer significant connectivity values. Still, a fair number of low, non-significant connectivity values has to be converted into edges in order to achieve the imposed *k*. This yields an unwanted emphasis on ‘irrelevant’ connections as they may be a mere by-product of the noise in neural data, in particular in networks with low average connectivity. On the contrary, for networks with large average connectivity, connections that are important may be ignored because including them would result in a too large average degree. In this way, applying a prescribed average degree *k* can modify the topology (Figure 2). To get an impression of how results are affected by the choice of threshold, graph measures might be calculated both as a function of a fixed threshold and with a fixed average degree [11], [16], [22].

#### Fixed edge density

An alternative but less common approach to define a threshold is to fix the network's edge density (also referred to as *wiring cost*), i.e. number of existing edges divided by the number of possible edges [41]. This approach is motivated by that fact that fixing the edge density implies fixing the probability for the existence of an edge in the case of Erdös-Rényi random networks. Choosing a fixed edge density can be a useful approach but it should be kept in mind that it also restrains the number of edges and hence may involve a modification of the topology under study as discussed in the previous paragraph. We note that for two networks with the same number of nodes this approach boils down to preserving the same average degree.

#### Weighted graph analysis: *N,k*-dependence remains

The transformation of connectivity values from a continuous to a binary scale entails many difficulties. While the binary scale clearly enhances contrast it also hides potentially important information as connectivity values below or above threshold may vary considerably between conditions. Weighted graph analysis seeks to preserve that information and also avoids all aforementioned issues related to the selection of an appropriate threshold. Therefore it has become more popular in recent studies [10], [23], [27]. Still, results for weighted graphs are not qualitatively very different from the unweighted network analysis [13], [38]. In principle, for weighted graphs one could eliminate connections that may be present by chance using a threshold to set all non-significant values to zero. Then the resulting graph exists of zeros and weights instead of mere zeros and ones. However, the importance of individual edges in the network scales with connectivity strength and non-significant connectivity values are therefore supposed to play a minor role in the network topology, also without thresholding. Unfortunately, using weighted graphs cannot solve the problem of the *N,k*-dependence of graph measures as two networks generally differ in connectivity values and their distribution. Just as differences in average degree for binary graphs, the differences in weights do influence graph measures.

### Approaches to correct for the *N,k*-dependence

If the topology of the network under study is known, one can immediately correct for size effects, at least when using graph measures that allow for a closed mathematical description (e.g., Table 1). If the topology is unknown, however, alternative corrections are required as illustrated numerically below. For all simulations we generated Erdös-Rényi random networks, ring lattices and Watts-Strogatz small-world networks with rewiring probability 0.1, while systematically varying *N* and *k*. All results are based on 200 repetitions.

#### Normalization by random graphs

**Figure 3:**
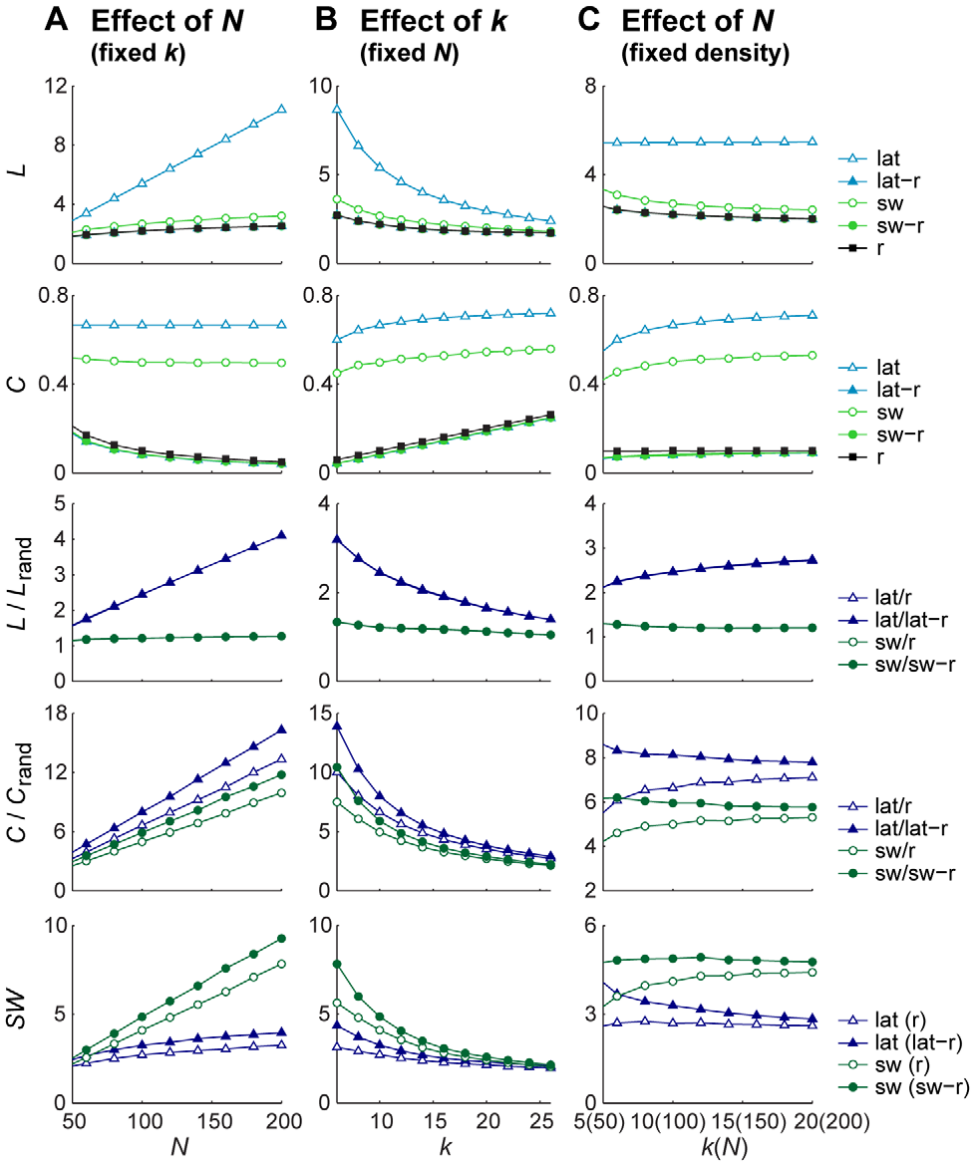
Normalized graph measures using random surrogates remain sensitive to network size and average degree. Path length (*L*) and clustering coefficient (*C*) normalized by dividing values from a lattice and small-world network (rewiring probability = 0.1) by those of random networks still depend on the network's number of nodes (*N*) and average degree (*k*) because their curves as function of *N* and *k* are specific for each type of network. Small-world networks (sw) fall in between lattices (lat) and random networks (r) and so the influence of normalization on the *N,k*-dependence is smaller compared to lattices. Since *L* for small-world networks is close to that of random networks, normalization improves the independence of *N* and *k* (more horizontal curves). By contrast, *C* is close to that of lattices and normalization introduces a bias that is larger compared to the non-normalized measure. Because of this, the small-world index (*SW*) is also greatly affected by *N* and *k*. There is little to no difference for Erdös-Rényi networks and random networks with the same degree distribution as the original network (lat-r, sw-r) and even coincide for *L*. The legend for *SW* indicates between brackets the type of random surrogates used in the calculation. **A**: Effects of changes in the number of nodes (*N*) while keeping the average degree constant (*k* = 10). **B**: Effects of changes in the average degree (*k*) while keeping the number of nodes constant (*N* = 100). **C**: Effects of changes in the number of nodes while keeping the edge density constant (0.1). Note that *k* now increases with network size. In this case, the sensitivity to network size is greatly reduced.

In searching for *N,k*-invariant analyses, several studies used random networks with the same number of nodes and connections as (bootstrapped) surrogates to normalize the corresponding graph measures (e.g., [18], [37], [44]). At first glance, that normalization appears elegant but as depicted in Figure 3, the trouble is again that the *N,k*-dependence of graph measures depends on network type. For fixed *k*, an increase in the number of nodes *N* has a larger effect on the path length *L* in regular networks (lattices) as opposed to random networks. Hence, the ratio *L*_lat_/*L*_rand_ depends on *N*, i.e. two lattices that differ in *N* will not display the same normalized values despite the equivalence of their topologies (Figure 3A). Similarly, if *N* is fixed, then the effect of adding edges and thus increasing *k* on the path length *L* is larger for a lattice than for a random network (Figure 3B). An even more pronounced difference between size effects in distinct topologies is found for the clustering coefficient (*C*), which is independent of *N* and *k* for lattices but not for random networks. There, the normalization introduces a bias that was absent in the non-normalized value. Equivalent findings hold for the *small-world index* (*SW*, see Figure 3, lower row), a graph measure commonly applied to assess small-world networks and relying on the here-discussed normalization [45]: it is defined as the ratio between normalized clustering coefficient and normalized path length. Strikingly, *SW* shows a strong *N,k*-dependence for small-world networks. Its linear dependence on *N* can be deduced from the analytical expressions in Table 1, as was shown by [45]. Without any corrections, the small-world index can hence not be used to compare the small-worldness of different empirical networks.

As mentioned earlier, in experimental settings the nodes often agree with recording sites and the number of connections is usually influenced by the researcher's choices. Figure S2 shows surface plots indicating that, when either *N* or *k* is fixed, a change in number of nodes or connections necessarily results in different normalized values for *L* and *C*. Only if *N* and *k* are both free to change (are independent variables) the same normalized value can be reached. Indeed when preserving the edge density *C* and *L* appear relatively insensitive to changes in size (Figure 3C). However, not all graph measures benefit from a fixed density since they show stronger dependence on network size (see below).

**Figure 4:**
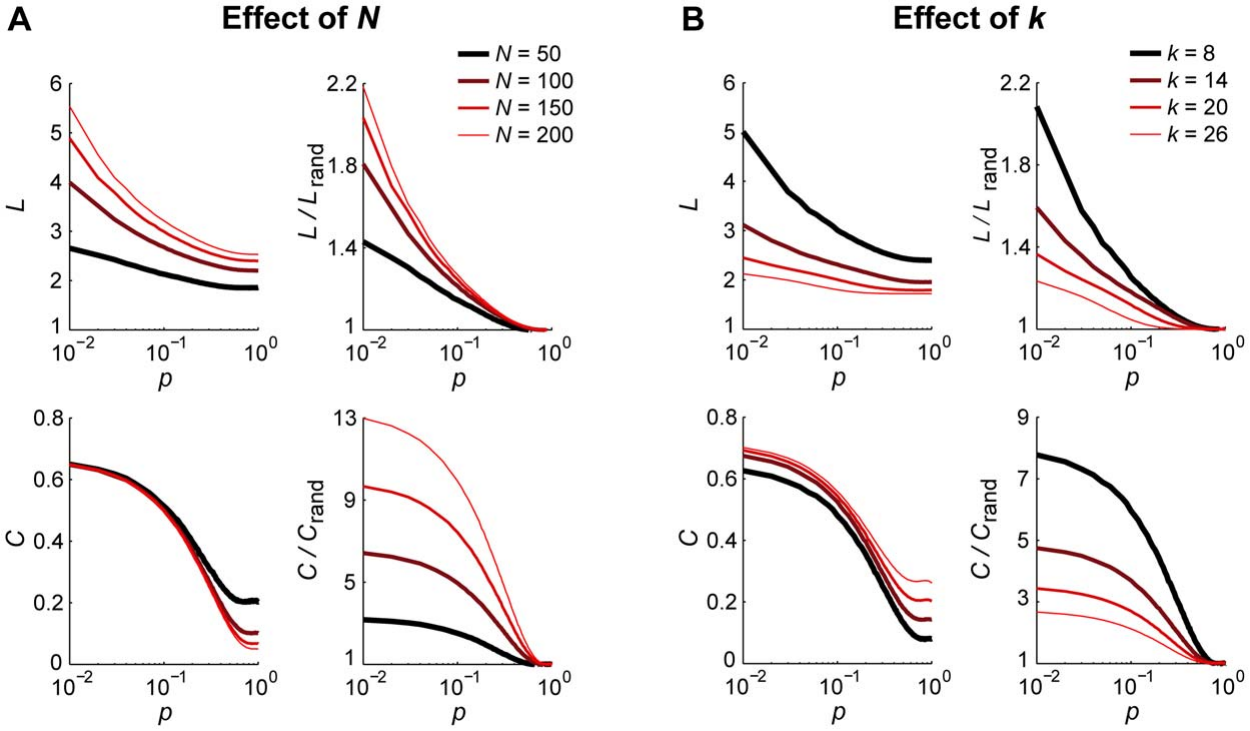
The sensitivity of small-world networks to size and average degree changes depends on rewiring probability. The dependence for normalized path length and clustering coefficient (*L*/*L*_rand_ and *C*/*C*_rand_ with Erdös-Rényi random networks) decreases with rewiring probability *p* (stronger resemblance to random networks). **A**: Effects of changes in the number of nodes (*N*) while keeping the average degree constant (*k* = 10). **B**: Effects of changes in the average degree (*k*) while keeping the number of nodes constant (*N* = 100).

One may argue that we seek the extreme cases as the *N*,*k*-dependence of *L* and *C* may differ most between lattices and random networks. Empirical networks often resemble small-world characteristics and, indeed, graph measures will probably have values that lie somewhere between those of a lattice and random network. In fact, since small-world networks are characterized by a path length close to that of random networks even for small rewiring probabilities, effects on normalized *L* are reduced. By contrast, however, the average clustering coefficient of small-world networks is close to that of a lattice and normalization by random networks introduces a larger *N,k*-dependence than seen for the non-normalized values. The small-world networks reported here had a rewiring probability of 0.1 (i.e. 10% random connections). In Figure 4 we show the *N,k*-dependencies of *C* and *L* for different rewiring probabilities. As expected, for low rewiring probabilities *L* is highly dependent on the number of nodes and edges, and much less so for high rewiring probabilities. The opposite is true for *C*. Remarkably, normalization by random networks only eliminates effects of *N* and *k* when the rewiring probability approaches 1, i.e. producing random networks. For lower rewiring probabilities, normalized *C* and *L* are still dependent on *N* and *k*. Hence, the exact bias introduced by differences in *N* and *k* depends on the rewiring probability.

Creating surrogates for normalization via plain randomization yields Erdös-Rényi random networks, which have a Poisson degree distribution. May it be so that by virtue of its shape the degree distribution introduces the undesired *N,k*-dependence outlined above? To avoid answering this question one can generate random surrogates that do not only match the original network's *N* and *k* but also have the same degree distribution [7], [13], [26], [30], [33]–[35]. We here used the algorithm described by Maslov and Sneppen [46] to generate these types of random networks and repeated all simulations but found little to no difference between these and plain random surrogates (see Figure 3). We note that for original empirical networks with an asymmetric degree distribution, the difference could become larger as for instance scale-free networks show different *N,k*-dependencies compared to Erdös-Rényi networks (cf. Table 1). However, we did not perform a systematic investigation of the *N,k*-dependence for scale-free networks because the preferential attachment models do not allow for a completely independent manipulation of *N* and *k*.

In addition to *L*, *C*, and *SW*, many other graph measures are also characterized by a *N,k*-dependence that depends on network topology. In the Figure S3 we illustrate this for the *number of hubs*, *maximum degree*, *synchronizability* and *central point dominance*. In fact, none of the here-discussed measures were entirely insensitive to changes in *N* and *k*, but several converged to a (almost) constant value with increasing number of nodes (*N*>200) and/or edges (*k*>25).

#### Normalization by the range of possible outcomes

**Figure 5:**
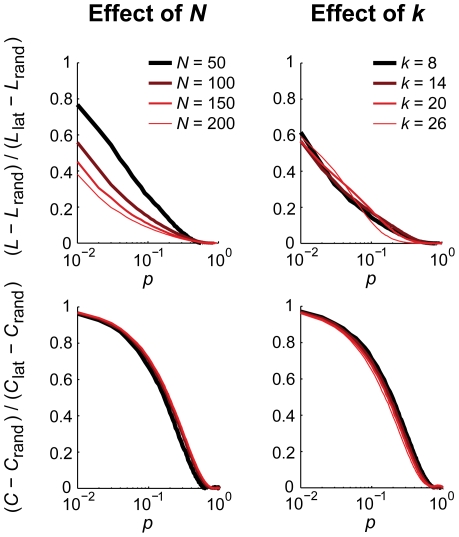
Normalization of graph measures by the range of possible values. Expressing path length (*L*) and clustering coefficient (*C*) as a ratio of the range of obtainable values (lattice - random) diminishes the sensitivity to changes in number of nodes *N* and average degree *k*. Only the path length remains largely sensitive to changes in *N* for a large range of rewiring probabilities *p* (the four curves do not coincide here). Shown are small-world networks with either a fixed *k* = 10 or a fixed *N* = 100.

Replacing random surrogates by other kinds of networks, e.g., lattices, will yield equivalent normalization problems. For instance, the aforementioned arguments around *L*_lat_/*L*_rand_ can be readily inverted, i.e. the ‘bias via random networks’ would be replaced by a ‘bias via lattices’ *L*_rand_/*L*_lat_ and the challenge regarding its *N,k*-dependence would persist. Nonetheless one may try to exploit the fact that many empirical networks appear to have small-world characteristics and design an according normalization. Given a small-world network with certain *N* and *k*, its values for *L* and *C* will be somewhere between those of a lattice and a random network. Hence one may express the observed *L* and *C* as a fraction of the range of possible obtainable values [3], [47], i.e.
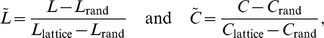
which may improve the insensitivity to changes in *N* and, in particular, *k* (Figure 5). As such, this approach might be useful for empirical networks that have a small-world structure. This, of course, limits the applicability of this normalization since the precise topology remains unknown. Other types of networks, however, may allow for similar approaches as the ranges of outcome values of, e.g., *L* and *C*, are bounded; see for instance the scale-free networks in Klemm and Eguíluz [48].

#### Estimating the *N,k*-dependence of graph measures

We argued repeatedly that if the *N,k*-dependence of graph measures for the network under study were known, one could immediately compensate for it, i.e. correct for a possible bias. However, the topology of empirical networks is the very characteristic that ought to be determined through analysis. Most likely, empirical networks do not have the topology of one of the archetypical networks listed in Table 1 leaving its baseline model unknown. Asymmetric degree distributions cannot be explained by the Watts-Strogatz small-world network (rewiring model) but by the Barabási-Albert scale-free network (preferential attachment model). The latter does not account for the large modularity, clustering coefficient and degree correlations. Obviously, a proper baseline model should be able to explain as many network features as possible, maximizing the likelihood that the empirical network was generated from that model. Approaches to estimate such a model will be discussed below but, so far, the lack of a model calls for alternative estimates of the network's *N,k*-dependence. To do so one may use sampled networks by randomly removing nodes or edges. However, this (and other forms of) down-sampling may readily change the network's topology [49]–[53] and thus bias, or at least influence, any estimate.

**Figure 6:**
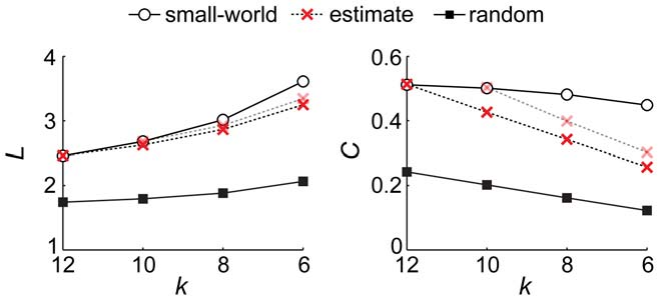
The random removal of edges introduces a bias towards random networks. Shown here is the estimation of the *k*-dependence for a small-world network (rewiring probability = 0.1). Edges were stepwise randomly removed from the original network and the path length (*L*) and clustering coefficient (*C*) were recalculated. This procedure was repeated 200 times, resulting in an average *k*-dependence estimate. We started this estimation both with a original network having *k* = 10 (dashed black line) and *k* = 12 dashed grey line). Estimates showed a deviation from the simulated *k*-dependence of the small-world network and depended on the average degree of the original network. Effects were smaller for *L* compared to *C* since values are already closer to those for random networks.

To illustrate this influence we estimated the *k*-dependence of *L* and *C* for a small-world network (Figure 6). We removed edges at random, by which *k* was stepwise decreased, and recalculated *L* and *C* at each step, i.e. for each sampled network. This process was repeated 200 times yielding a mean *k*-dependence of path length and clustering coefficient. Both measures deviated from the expected values of small-world networks and tended toward those of a random network. Moreover the *k*-dependencies depended on the size of the original network, i.e. on the number of edges that had to be removed in order to reach a specific average degree. Effects were relatively small for *L* because the path length of a small-world network is typically close to that of a random network. That is, for graph measures not close to that of a random network, randomly removing edges does not lead to a correct estimation of the *k*-dependence (i.e. the true *k*-dependence for the investigated network type).

We note that the random removal of edges from the small-world network does not affect the ratio between local and long-range connections (i.e. the rewiring probability). Of the total number of edges removed, the fraction of removed long-range connections will be *p* as well. For example, a network with *N* = 100, *k* = 10 and *p* = 0.1 containing 900 local connections and 100 long range connections, removing 200 edges will on average affect 180 local connections and twenty long range connections, resulting in a network with *N* = 100, *k* = 8 and *p* = 0.1. However, the analytical *k*-dependence for a small-world network with *k* = 8 will be based on a lattice connected to its four neighbors on each side, while the estimated relation is based on a lattice with connections to five neighbors. If local edges are removed, the clustering between direct neighbors will be underestimated and, therefore, the network becomes more random.

### A fling with social sciences: comparing social networks

The problem of comparing networks with different size and connectivity density has been recognized in other disciplines. We here highlight social networks where the application of graph theory has a long tradition ([54], [55], and see e.g., [56], [57] for recent reviews). Social networks may significantly vary in number and type of connections and size and type of studied population (e.g., friendships among high school students, advice seeking among company employees, collaborations between movie actors, agonistic encounters for certain animal species, etc.). We briefly sketch four methods that have been considered useful when comparing networks, although, strictly speaking, all these methods are also not entirely insensitive to differences in *N* and *k* (see [56], [58], [59], and references therein).

#### Distances and correlations between graphs

The most direct way of comparing networks is to assess their distances [60] or their more common covariance and correlation [56]. Distances between graphs typically build on the Hamming distance, which is a very general measure and forms a metric on the space of graphs, be they directed or undirected. The Hamming distance gives the number of elements of two graphs ***y***_1_ and ***y***_2_ with adjacency matrices ***A*** ^(1)^ and ***A*** ^(2)^ that disagree, or more formally
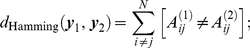
the 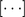 notation here reflects an indicator function that is equal to one if its argument is true and zero otherwise. The Hamming distance may also be viewed of as the number of addition/deletion operations required turning the set of edges of ***y***_1_ into that of ***y***_2_. If nodes are interchangeable (i.e. their locations are irrelevant and hence their order in the adjacency matrix may be different for two networks) the Hamming distance may yield spurious results, which led Butts and Carley [60] to formulate the *structural distance*. The structural distance between ***y***_1_ and ***y***_2_ is defined as
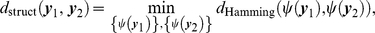
where 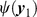 and 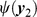 denote node permutations of ***y***_1_ and ***y***_2_ out of the corresponding sets of all accessible permutations, i.e. 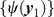 and 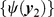, respectively.

In order to define *d*_Hamming_ and, hence, *d*_struct_, the adjacency matrices must match in size, i.e. the two networks have the same number of nodes, which greatly limits applicability. In the specific case that the networks agree in the number of nodes, then the distance trivially scales with *N* and *k*. As an alternative to the distance one may define the *covariance* between the graphs ***y***_1_ and ***y***_2_ as
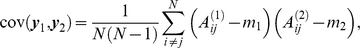
where *m*_1_ and *m*_2_ are the means of the respective adjacency matrices ***A*** ^(1)^ and ***A*** ^(2)^, e.g.,
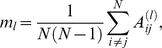
which equals the edge density of the network in the case of unweighted graphs; recall that the self-adjacencies *A_ii_* vanish by construction. The corresponding graph correlation is readily obtained by normalization via the individual variances in terms of
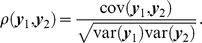
In words, using the graph covariance the existence of a particular edge *i*↔*j* is compared between adjacency matrices. For unweighted, i.e. binary graphs, a correlation of 1 will be obtained if and only if all edges in ***y***_1_ also exist in ***y***_2_. By contrast, a correlation of −1 will be obtained if and only if the two graphs are completely mirrored, i.e. all edges in ***y***_1_ do not exist in ***y***_2_ and vice versa.

A similar approach has been discussed by Costa and colleagues [61], who also employed a normalization to correct for large baseline correlations in sparse binary matrices. Expressing the number of coinciding ones and zeros in ***A*** ^(1)^ and ***A*** ^(2)^ as a ratio of the total number of ones and zeros in ***A*** ^(1)^ in terms of
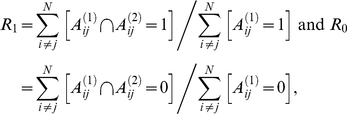
the geometrical average between the ratios 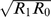 could be used as a distance measure.

As for the distance measures, the two adjacency matrices must again have the same size *N*. Since this restriction does not apply for the mean value, a difference in the number of connections causes the maximum correlation to be always strictly smaller than 1 (e.g., since edges are compared pair-wise, there is at least one edge that exists in the graph with the highest mean that does not exist in the graph with the lowest mean). Or, if the sum of the number of edges in both graphs does not equal the amount of possible edges, *N*(*N*−1), then the minimum correlation is strictly smaller (less negative) than −1. Fortunately, the maximum and minimum obtainable correlations can be easily calculated by reordering the values in the adjacency matrices to obtain as many overlapping or non-overlapping edges as possible offering the possibility for normalization with respect to the maximum correlation.

#### Comparison with size effects in a baseline model

To go beyond the mere comparison of adjacencies, Anderson and others [58] proposed to contrast networks by comparing the observed difference in a graph measure *g*, i.e. 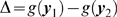, with the difference predicted by a *baseline model*, which is assumed to mimic the main characteristics of both empirical networks. One generates a random realization of the baseline model that agrees in size *N* and average degree *k* with the first empirical network, and another realization that matches the second empirical network in *N* and *k*. From the two simulated networks we obtain a difference in the graph measure 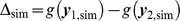. Repeating this process several times leads to a probability distribution 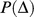, which allows for defining a 95% confidence interval of the probability that the observed difference has been caused by changes in *N* and *k*. Although this method is theoretically very appealing, it is difficult to apply because the baseline model of the empirical networks is usually unknown. Moreover, baseline models may differ from the to-be-compared networks, e.g., between subjects or conditions, which certainly complicates the estimation of a common probability distribution.

We show these difficulties by investigating examples of anatomical networks in the human brain reported by Hagmann and colleagues [10]. We considered a Watts-Strogatz small-world network with fixed rewiring probability as baseline model and used the small-world index *SW* as graph measure *g*, which we found to be very sensitive to changes in *N* and *k* (see above). The empirical networks contained weighted, undirected anatomical connections (axonal pathways obtained from diffusion spectrum imaging) between 998 regions of interest covering the entire cortex (data were obtained from the authors of [10]). The 998-node network, graph 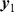, was averaged over all five subjects. To illustrate size effects we further averaged fiber densities within and between the areas of a 66-node parcellation scheme yielding a corresponding 66-node network, graph 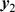 as was also done in the original paper. For these two networks we found the small-world indices to be *SW* = 7.84 and 1.84, for the 998 and 66-node networks respectively, resulting in 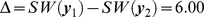.

Next, the rewiring probabilities of the two networks were individually estimated by simulating small-world networks with the same weights as the original networks. This was done by randomly placing the weights of the empirical networks in a ring lattice and subsequent rewiring to create long-distance connections. The rewiring probability was determined through least squares optimization of the respective *SW* [45]. The first problem was that the optimization revealed different rewiring probabilities for the two networks, namely 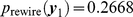 for *N* = 998 versus 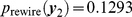 for *N* = 66. That is, strictly speaking the two networks had different baseline models rendering a subsequent comparison that builds on one of the models doubtful. Nonetheless we proceeded with the rewiring probabilities at hand and generated for both of them different sets of small-world networks with 998 and 66 nodes (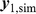 and 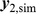 with 2000 realizations each), and computed the distribution of differences 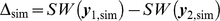.

For *p*_rewire_ = 0.1293 and 0.2668 we found the corresponding confidence interval as 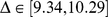 and 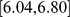, respectively, implying that in both cases the observed difference cannot be exclusively admitted to mere size effects, i.e. the baseline model may be altered and, hence, topology is not necessarily preserved. The mismatch for *p*_rewire_ = 0.1293 is even stronger because changing the rewiring probability has a larger effect on *SW* in the case of large networks than for small networks – recall that *p_rewire_* = 0.1293 was determined from *N* = 66, that is, in this case we used the baseline model of the smaller network to mimic the larger one.

It should be evident that a proper interpretation of this comparison requires great confidence in the validity of the underlying baseline model. In our example the two networks might have differed in topology. We note that down-sampling is indeed known to affect network characteristics [49]–[53]. And even if the networks do have the same topology one may question whether a Watts-Strogatz rewiring model is actually the proper baseline (e.g., how about the networks' degree distribution, modularity, etc.?). Of course, some uncertainty about the baseline model will persist. After all, if the empirical network would perfectly agree with a known (theoretical) baseline model, its *N,k*-dependence might directly be accessible, i.e. one can generate an according invariance by algebraic means rendering the here-discussed problems immaterial.

#### Exponential random graph models

In the absence of an accurate model description one may estimate the likelihood that a certain parameterized model provides reasonable if not proper estimates. Also known as ‘p*’ models, exponential random graph models are models that describe the probability of a number of statistics *g_μ_* to be present in graph ***y*** [62]–[64]. These statistics may be any of the here-discussed average graph measures like *k*, *C*, *L*, *SW*, the degree distribution, the Hamming distance to another network, but also other structural properties like certain motifs (e.g., edges, stars, triangles; see also below and Figure S4). The incidences of the statistics are given by a set of parameters *θ_μ_* that can be determined via maximizing the likelihood of the model [65]. This maximum likelihood estimate agrees with a conventional maximum entropy approach constraint by the aforementioned statistics where the parameters become Lagrange parameters [66]. Put differently, exponential random graph models are baseline models of a network by assuming that all realized networks are maximally ‘random’ given the average values of their statistics [56]. More formally one may write
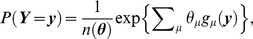
where 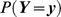 denotes the probability that the empirical network, i.e. graph ***y***, belongs to the class of networks ***Y*** that are characterized by the set of parameter values ***θ*** = *θ*_1_,*θ*_2_,*θ*_3_, …. As said, these parameters *θ_μ_* are varied in order to specify the model that is most probable to underlie ***y***, i.e. they weigh the presence of the statistics *g_μ_*. The normalizing constant *n* depends on the parameter values ***θ*** and ensures that the distribution of ***Y*** describes a probability (i.e. its integral equals one). Finding parameter values ***θ*** can be realized via Markov chains or other maximum (pseudo-)likelihood methods (see e.g., [65] for details). If a certain *θ_μ_* turns out to be large and positive, the corresponding statistics *g_μ_* can be considered a major ingredient of the empirical network under study. If *θ_μ_* is negative, the corresponding statistic is less prevalent than might be expected by chance. In both cases a significant *θ_μ_* reveals an important deviation from the null model (random network) and, hence, contributes to the network's topology. In order to compare networks, model fits are obtained for all networks using a common set of statistics – see below. Once the optimal parameter values have been determined for all empirical networks, either parameter values could be directly compared between networks, or when dealing with multiple networks, the resemblance between networks could be investigated by comparing predicted probabilities based on parameter values from other networks [59]. In the following paragraph we illustrate the application of exponential random graph models by contrasting it with motif counting.

#### Counting motifs

To detect the primary building blocks of empirical networks Sporns and colleagues [3], [67] suggested to simply count different motifs and evaluate their relevance through a post-hoc bootstrapping statistics. Put differently, histograms (‘frequency spectra’) of motifs are compared with those of random surrogates by which the probability for the presence of a certain motif can be estimated including a corresponding significance value. As such this approach is closely related to the afore-discussed comparison of Anderson and others [58] when the graph measure is restricted to the occurrence of a motif and the random network is chosen as baseline model; in [67] the networks were also contrasted with a lattice but this comparison is ‘only’ descriptive as no statistics could be performed. Connected motifs were categorized by conventional graph theoretical means, i.e. ordered by their number of nodes *N* into dyads (*N* = 2; for directed graphs there are 2 distinct connected dyads: asymmetric and mutual), triads (*N* = 3; for directed graphs there are in total 16 triads but only 13 are connected), et cetera. This yielded structural motifs, which can contain a set of so-called functional motifs. For instance, a mutual dyad contains two asymmetric dyads, a mutually connected triangle may contain 2-stars (in-, out-, mixed-stars), cyclic triples, and so on. Put differently, a functional motif can appear via different structural motifs, i.e. the structural motifs form the base on which functional motifs may emerge. Interestingly, it has been hypothesized that brain networks maximize both the number and the miscellany of functional motifs, while the stock of structural motifs remains small – note that by construction the latter must be smaller. In fact, when constraining a random network model by the functional motif number, network topologies have been simulated that resemble various graph measures of the empirical networks under study [67].

Counting motifs is closely related to the exponential random graph models, at least when restricting to the identification of motif fingerprints [3]. However, the first requires a post-hoc statistical assessment whereas for the latter statistical testing is immanent so that result may readily differ. We investigated this contrast by optimizing several exponential random graph models in 10 iterative steps each with 10^5^ networks that were randomly drawn from the distribution on the set of all networks; optimization was realized via a Monte Carlo Markov chain methods using the Metropolis-Hastings algorithm with 10^3^ proposals, see the Statnet software package [68], [69] for more details. In the exponential random graph models the sign of the parameter values is determined relative to a random network with edge probability of 0.5 whereas Sporns and Kötter [67] chose the edge probability so that the degree of their random graph matched with the empirical network. We only used the average degree of the empirical network to constrain the Monte-Carlo optimization.

**Figure 7:**
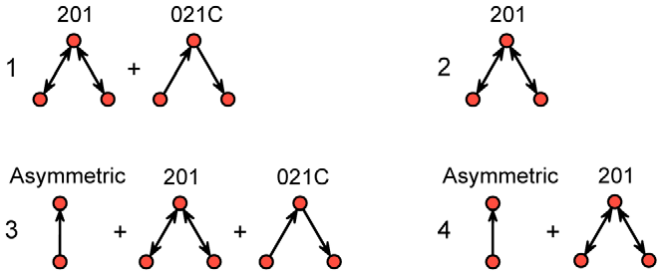
Graphical representation of our four exponential random graph models. The triads are given conventional numbering; see Figure S4 for a complete overview of all possible dyads and triads.

We used data of the anatomical connections in the macaque visual cortex consisting of a directed, unweighted graph with 30 nodes, and 311 edges (available at http://www.brain-connectivity-toolbox.net). According to Sporns and Kötter [67], in this network the frequency of only five structural motifs appeared significantly increased when optimizing a random network that was constrained by the number of estimated functional motifs (see Figure 3B and Table 4 in [67]). Since we did not intend to replicate these results to all extent but rather want to highlight differences between methods we here only considered motifs with up to three nodes, which left a single triad, namely triads census 201 ([59], [70], see also Figure 7 and S4), i.e. the mutually connected two-path (ID = 9 in [67]). Interestingly, however, the counts of six other motifs with three nodes where significantly decreased but not further discussed in [67], although the reduced frequency of such mostly directed motifs is certainly as interesting as the increased count of triad census 201. The combination of these in total seven motifs led to the first exponential random graph model that was unfortunately degenerate [71]. We therefore reduced the model a priori to
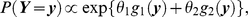
with *g*_1_ referring to triad 201 and *g*_2_ to 021C (see Figure 7). Optimizations revealed that in this model #1 both parameters were significant and, in agreement with [67] (Table 2, z-scores of the simulation with random networks) *θ*_1_ was positive and *θ*_2_ negative. We re-analyzed this further aiming for an optimal model fit by means of minimizing Akaike's information criterion (AIC) as heuristic for model selection [72]; the AIC-values were determined via the approximated log-likelihood that the empirical network was drawn from the distribution of the corresponding exponential random graph model with optimized parameters ***θ***.

**Table 2:** Parameter estimates and model performance for the exponential random graph models.

Model	Motif	*θ_μ_*	ΔAIC	Akaike weights	Explained deviance (df)
#1	201	0.07223***	88	0.00	390 (2)
	021C	−0.60012***			
#2	201	0.237444***	286	0.00	190 (1)
#3	asym	−1.16814***	0	1.00	480 (3)
	201	−0.26603**			
	021C	−0.03130			
#4	asym	−2.23637***	22	0.00	457 (2)
	201	−0.12503***			

Significance levels: *<0.05, **<0.01, ***<0.001. Akaike weights were calculated according to [81]. The total degrees of freedom (df) equals the number of possible edges, *n*(*n*−1) = 870. The null deviance was 1206 (870).

First, we compared this to the model of Sporns and Kötter [67], which we here called model #2 formalized as
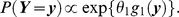
Again, *θ*_1_ was significant and positive but the model yielded a larger AIC-value rendering this single motif model less accurate than model #1 (Sporns and Kötter's choice for this model also included comparison with other empirical networks, so that for the macaque visual cortex alone it might not perform optimal). Next, we supplemented model #1 by a simple, asymmetric dyad (*g*_0_)
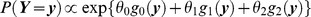
and found that this model #3, in fact, had a very low AIC-value but not all parameters reached significance. Therefore, we tested this further against another reduced model #4 that contained only two statistics, namely,
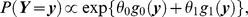
which provided a less optimal fit when looking at its AIC value. All results are summarized in Table 2 and for the goodness-of-fit assessments see Figure S5.

In our example adding more statistics often led to so-called ‘model degeneracy’; for instance, when for certain parameter values ***θ***, the exponential random graph model yields a distribution, in which only a handful of graphs have nonzero probability, which renders optimization unstable and model fits unreliable [73]. A detailed discussion about this important limitation of the (blind) applicability of exponential random graph models is, however, beyond the scope of the current illustration, so that we rather refer to the existing literature [71], [74]–[77]. Here it appears that the combination of counting motifs and according statistical results can certainly provide starting points for choosing proper exponential random graph models.

Because motif counts as well as exponential random graph models are based on probability estimates, they appear very useful to compare networks of different size. With increasing connectivity density, however, some motifs might become more likely to occur than others. For example, in a sparsely connected network isolated nodes and asymmetric connections will dominate the topology, whereas more complicated ones (e.g., 3-stars) are more likely in a densely connected graph. To avoid this ‘bias’ one could try to extend the set of statistics with other graph properties like the nodal degree or measures related to distance and centralization. As mentioned above, exponential random graph models do allow for incorporating further statistics like the average graph measures (e.g., *k*, *C*, *L*, *SW*) but also the degree distribution and so on. As such the exponential random graph model approach appears more general and more adjustable than mere motif counting and subsequent categorization. The restriction to motifs, however, yields readily interpretable results in terms which combination of structural building blocks (i.e. motifs) yields which topological characteristic.

## Discussion

The application of graph theory can provide very valuable insights in the structural and functional organization of neural networks. Its use for comparing network topologies, however, is not without challenges. The major difficulty arises from the fact that graph measures depend on the number of nodes and edges in a way that is specific for the type of network topology. To our best knowledge, satisfactory methods to correct for size and connectivity density dependent effects do not exist, yet. Because experimental data yield networks whose topologies do not necessarily agree with the frequently discussed archetypical networks (e.g., lattices, small-world with a certain rewiring probability, or fully random networks), it remains tricky to estimate how graph measures are influenced by changes in *N* and *k* for the empirical network. That is, discriminating between differences in graph measures in the two networks that are due to their *N*,*k*-dependence or caused by ‘true’ effects of experimental conditions is not easy, if at all possible. Not only can significant effects be introduced by the *N*,*k*-dependence, true effects may also be masked due to opposite effects. We have shown that some methods are less sensitive to changes in *N* and *k* than others but there is a clear need for a further search for proper measures.

The sensitivity varies across measures, with specifically large effects for the commonly used measures *C*/*C*_rand_ and the small-world index *SW*, contrasting less affected measures as *L*/*L*_rand_ and the non-normalized clustering coefficient *C*. Effects were particularly large for small-size networks like the ones typically obtained from M/EEG recordings, region-of-interest approaches, or physiologically tracked networks. The normalized path length and the non-normalized clustering coefficient, for instance, are less susceptible to changes in network size. We illustrated these *N,k*-dependencies for network types with a symmetrical degree distribution. However, there is no reason to believe that effects will disappear for networks with a more realistic, asymmetric degree distribution.

Despite all these difficulties we would like to point out that graph theory can be very beneficial not only for determining network topologies but also for pinpointing other important (local) network features that can be compared between networks, e.g., regions of interest by detecting the location of hubs or the occurrence of communities (modules) in the network. Likewise, the distribution of nodal values for a particular graph measure can be very insightful. For example, one may ask if the nodal clustering coefficients are uniformly distributed, whether there are a few nodes that display exceptional large values, or if the shape of the distribution differs between networks. In a similar way, the average degree, path length, and other measures can be addressed yielding respective values per individual node. In order to compare between conditions and/or subjects, a normalization of the distributions will most certainly be necessary.

Interestingly, the problem of comparing networks has not received much attention in neuroscience literature. This does not mean that graph analysis has not been used to compare network topologies. On the contrary, it has gained a lot of interest in recent years and is increasingly being applied in both functional and anatomical studies, which makes it even more important to recognize the numerous pitfalls involved in comparing networks. Without doubt, the application of graph analysis to experimental data is a less established research field than its mathematically-driven, theoretical counterpart. Analytic expressions are often only valid in the limit of large graph size but most empirical studies on brain networks use graphs with *N*<200 (some exceptions include [10], [12], [16], [78]). All graph measures investigated here through simulations showed a *N,k*-dependence that cannot be neglected in this range. By and large, empirical studies rarely account for *N,k*-dependent biases when estimating graph measures. As said, we are convinced that graph theory is a valuable tool for analyzing brain networks but its use has its challenges asking for great care when interpreting results.

To compare empirical networks, choosing equal size and density has become more popular so that differences in graph measures appear solely through structural changes. However, this can only be achieved by taking a fixed number of nodes and imposing a desired average degree by adjusting the binary threshold. Obviously, this has the disadvantage of manipulating the empirical network by over- or underrating connections [79]. Hence, either applying a fixed *N* and *k* or comparing networks with different *N* and *k* will lead to a certain bias. As both approaches appear complementary it might be useful to consider both networks with varying and fixed degree, and, if possible, supplement this with the several alternative methods summarized in this paper. The specific research question at hand may channel the proper decision for the most appropriate measure and the most reliable approach for comparison. One should not forget that differences in overall connectivity between subjects and conditions could be a very profound experimental result instead of a mere confounder. In any case, potential size-induced biases in graph measures should not be underestimated, in particular in the case of reasonably small networks, even if size differences between networks are small. These subtleties require great accuracy when applying methods and great caution when interpreting results.

## Supporting Information

Text S1 Formal definitions of graph measures.
(0.05 MB PDF)

Figure S1 Graph measures depend on network topology but also on network size and average degree. Shown here are Erdös-Rényi random networks with corresponding path lengths (*L*) and clustering coefficients (*C*). A: Increasing the number of nodes (*N*) results in an increase in *L* and a decrease in *C*. B: Increasing the average degree (*k*) results in a decrease in *L* and an increase in *C*. C: Increasing the number of nodes while preserving the same edge density keeps *C* and *L* approximately constant.
(0.80 MB TIF)

Figure S2 3D surface plots of the relation between changes in network size and average degree. Increasing only the number of nodes (*N*) or average degree (*k*) introduces a change in (normalized) path length (*L*) and clustering coefficient (*C*). Same values can only be reached by adjusting both the number of nodes and average degree at the same time. Shown here for a small-world network with a rewiring probability of 0.1. Contour lines are plotted on top for better visualization.
(3.76 MB TIF)

Figure S3 Sensitivity of other graph measures to changes in network size and average degree. The path length, clustering coefficient and small-world index in the main text's Figure 3 are not the only graph measures showing *N*,*k*-dependencies that are specific to the type of network. The number of hubs (*NHUBS*) scales linearly with the number of nodes in a network. The occurrence of hubs in lattices here for the right plot results from the fact that the average degree is adapted for each *N* to preserve the fixed edge density. The *k*-values on the x-axis are rounded, the real values are non-integers leaving some nodes to have one edge extra than others and as a consequence are classified as ‘hubs’. The maximum degree (*MAXD*) naturally increases with the number of edges in the network. Synchronizability (*S*) and central point dominance (*CPD*) mainly depend on the network's average degree, so that a fixed wiring cost cannot reduce the independence from changes in network size. For all measures with *N*,*k*-dependencies that are specific to the type of network, normalization by random graphs will by construction lead again to *N*,*k*-dependent measures. Networks here either have a fixed average degree *k* = 10, fixed number of nodes *N* = 100 or a fixed edge density = 0.1. lat, lattice; lat-r random network with uniform degree distribution; sw, small-world network with a rewiring probability of 0.1; sw-r, random network with same degree distribution as sw; r, Erdös-Rényi random network. Exact definitions of all measures can be found in Text S1.
(5.98 MB TIF)

Figure S4 Examples of directed network motifs. Shown are all possible dyads, triads and examples of k-instars, k-outstars and k-cycles. The number of possibilities rapidly increases for motifs with more than 3 nodes. These and other motifs could in principle all be used for both exponential random graph models and motif counting.
(5.96 MB TIF)

Figure S5 Goodness-of-fit diagnostics for the four exponential random graph models. The red lines represent the statistics of the observed network. The distribution of 100 networks simulated with the estimated parameter values of the model is indicated by the boxplots. The grey lines represent 95% confidence intervals. For the order of the triad census, see Figure S4.
(6.99 MB TIF)
