# Supplementary material for: Comparing Brain Networks of Different Size and Connectivity Density Using Graph Theory
Source: PLoS One. 2010 Oct 28;5(10):e13701. doi: 10.1371/journal.pone.0013701 (PMC2965659; doi:10.1371/journal.pone.0013701)
Supplement: Text S1 — Formal definitions of graph measures. (0.05 MB PDF) [file pone.0013701.s001.pdf]

Formal definitions of graph measures

We employed several commonly used measures that are listed below. We did not include *assortativity* because of the symmetric degree distributions we used for the small-world and Erdös-Rényi networks.

Average degree (k)

The degree of node *i* is defined as the sum of the row elements *j* in the adjacency matrix ***A*** containing binary values that represent the network's connectivity

$$k_i = \sum_j^N A_{ij}$$

and the average degree *k* is the average over all nodal degrees

$$\langle k \rangle = \frac{1}{N} \sum_i^N k_i = \frac{1}{N} \sum_{i,j}^N A_{ij} \ .$$

Edge density

The edge density represents the fraction of existing edges in the network out of the total number of possible edges

$$m = \frac{1}{N(N-1)} \sum_{\substack{i,j \\ i \neq j}}^N A_{ij} \ .$$

Characteristic path length (L)

L represents the average minimum number of edges between any two points in the network. To account for isolated nodes, we used the so-called global efficiency

$$E = \frac{1}{N(N-1)} \sum_{\substack{i,j \\ i \neq j}}^N \frac{1}{d_{ij}},$$

where d_{ij} is the minimum number of edges between nodes *i* and *j*, and defined *L* as the harmonic mean $L = 1/E$.

Average clustering coefficient (C)

The average clustering coefficient refers to the probability that neighbors of a node are also connected. It measures the occurrence of clusters in the network by means of

$$C = \frac{1}{N} \sum_i^N \frac{2n_i}{k_i(k_i - 1)},$$

where n_i represents the number of existing edges between neighbors of node i.

Small-world index (SW)

The small-world index has often been used to indicate the presence of a small-world network [1-6]. It is defined as

$$SW = \frac{C / C_{\text{rand}}}{L / L_{\text{rand}}},$$

where C_{rand} and L_{rand} are the clustering coefficient and path length of random networks with the same number of nodes and edges and often also the same degree distribution. Since small-world networks are characterized by $C \gg C_{\text{rand}}$ and $L \approx L_{\text{rand}}$, the ratio becomes $SW \gg 1$.

Number of hubs (NHUBS)

We assigned nodes as hubs when their nodal degree exceeded the average degree of the network

$$NHUBS = \sum_{i}^{N} \left[k_i > \langle k \rangle \right].$$

Maximum degree (MAXD)

The largest nodal degree defines the maximum degree of the network in terms of

$$MAXD = \max_i \left[k_i \right].$$

Synchronizability (S)

S describes the network's capacity to synchronize [see, e.g., 7,8] and is calculated from the eigenvalues of the graph's Laplacian matrix $\boldsymbol{\Lambda}$

$$\boldsymbol{\Lambda} = \boldsymbol{D} - \boldsymbol{A},$$

where $\boldsymbol{D}$ represents a diagonal matrix containing the nodal degrees. The synchronizability is defined as the ratio between the first non-zero eigenvalue λ_2 and the largest eigenvalue λ_{max} of $\boldsymbol{\Lambda}$, that is,

$$S = \frac{\lambda_2}{\lambda_{\text{max}}}.$$

Central point dominance (CPD)

The central point dominance is a measure that describes the distribution of betweenness centrality scores of nodes B_i, i.e. the fraction of shortest paths from node p to node q that runs through node i. It was introduced by Freeman [9] and defined as follows

$$CPD = \frac{1}{N-1}\sum_{i}^{N}\left(B_{\max} - B_i\right),$$

where

$$B_i = \frac{2}{(N-1)(N-2)}\sum_{i \neq j}^{N}\frac{\sigma(p,i,q)}{\sigma(p,q)}.$$

The number of shortest paths between node p and node q that run through node i is indicated here by $\sigma(p,i,q)$, the number of total shortest paths between node p and q by $\sigma(p,q)$.

References

1. Bassett DS, Bullmore E, Verchinski BA, Mattay VS, Weinberger DR, et al. (2008) Hierarchical organization of human cortical networks in health and schizophrenia. J Neurosci 28: 9239-9248.
2. Achard S, Salvador R, Whitcher B, Suckling J, Bullmore E (2006) A resilient, low-frequency, small-world human brain functional network with highly connected association cortical hubs. J Neurosci 26: 63-72.
3. de Haan W, Pijnenburg YAL, Strijers RLM, van der Made Y, van der Flier WM, et al. (2009) Functional neural network analysis in frontotemporal dementia and Alzheimer's disease using EEG and graph theory. BMC Neurosci 10: 101.
4. Liu Y, Liang M, Zhou Y, He Y, Hao Y, et al. (2008) Disrupted small-world networks in schizophrenia. Brain 131: 945-961.
5. Smit DJA, Stam CJ, Posthuma D, Boomsma DI, de Geus EJC (2008) Heritability of "small-world" networks in the brain: A graph theoretical analysis of resting-state EEG functional connectivity. Hum Brain Mapp 29: 1368-1378.
6. Humphries MD, Gurney K (2008) Network 'small-world-ness': A quantitative method for determining canonical network equivalence. Plos One 3: e0002051.
7. Bassett DS, Meyer-Lindenberg A, Achard S, Duke T, Bullmore E (2006) Adaptive reconfiguration of fractal small-world human brain functional networks. Proc Natl Acad Sci U S A 103: 19518-19523.
8. Nishikawa T, Motter AE, Lai YC, Hoppensteadt FC (2003) Heterogeneity in oscillator networks: Are smaller worlds easier to synchronize? Phys Rev Lett 91: 014101.
9. Freeman LC (1977) A set of measures of centrality based on betweenness. Sociometry 40: 35-41.
