# Supplementary figures and images for: Comparing Brain Networks of Different Size and Connectivity Density Using Graph Theory
Source: PLoS One. 2010 Oct 28;5(10):e13701. doi: 10.1371/journal.pone.0013701 (PMC2965659; doi:10.1371/journal.pone.0013701)

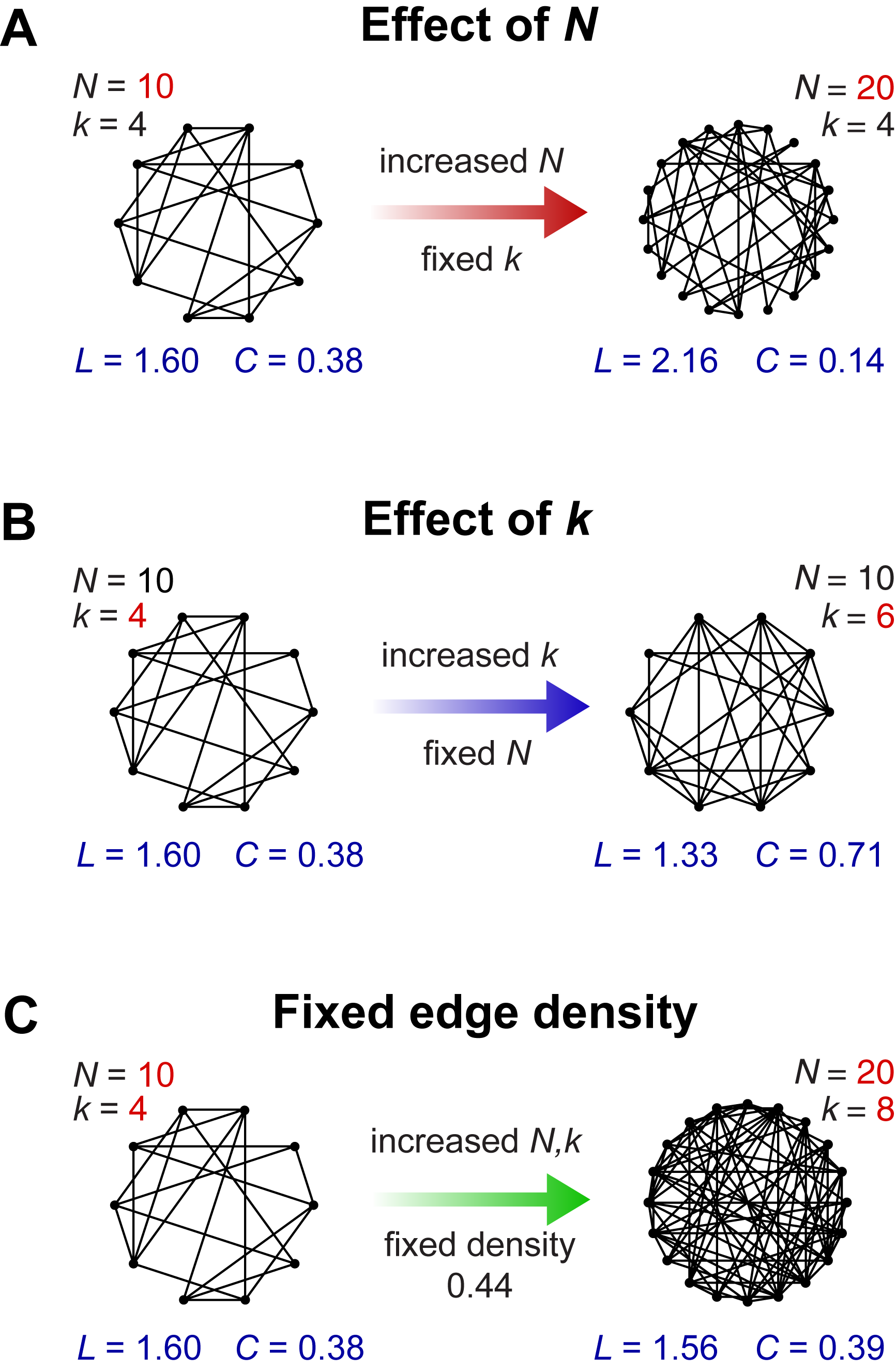

Supplement: Figure S1 — Graph measures depend on network topology but also on network size and average degree. Shown here are Erdös-Rényi random networks with corresponding path lengths (L) and clustering coefficients (C). A: Increasing the number of nodes (N) results in an increase in L and a decrease in C. B: Increasing the average degree (k) results in a decrease in L and an increase in C. C: Increasing the number of nodes while preserving the same edge density keeps C and L approximately constant. (0.80 MB TIF) [file pone.0013701.s002.tif]

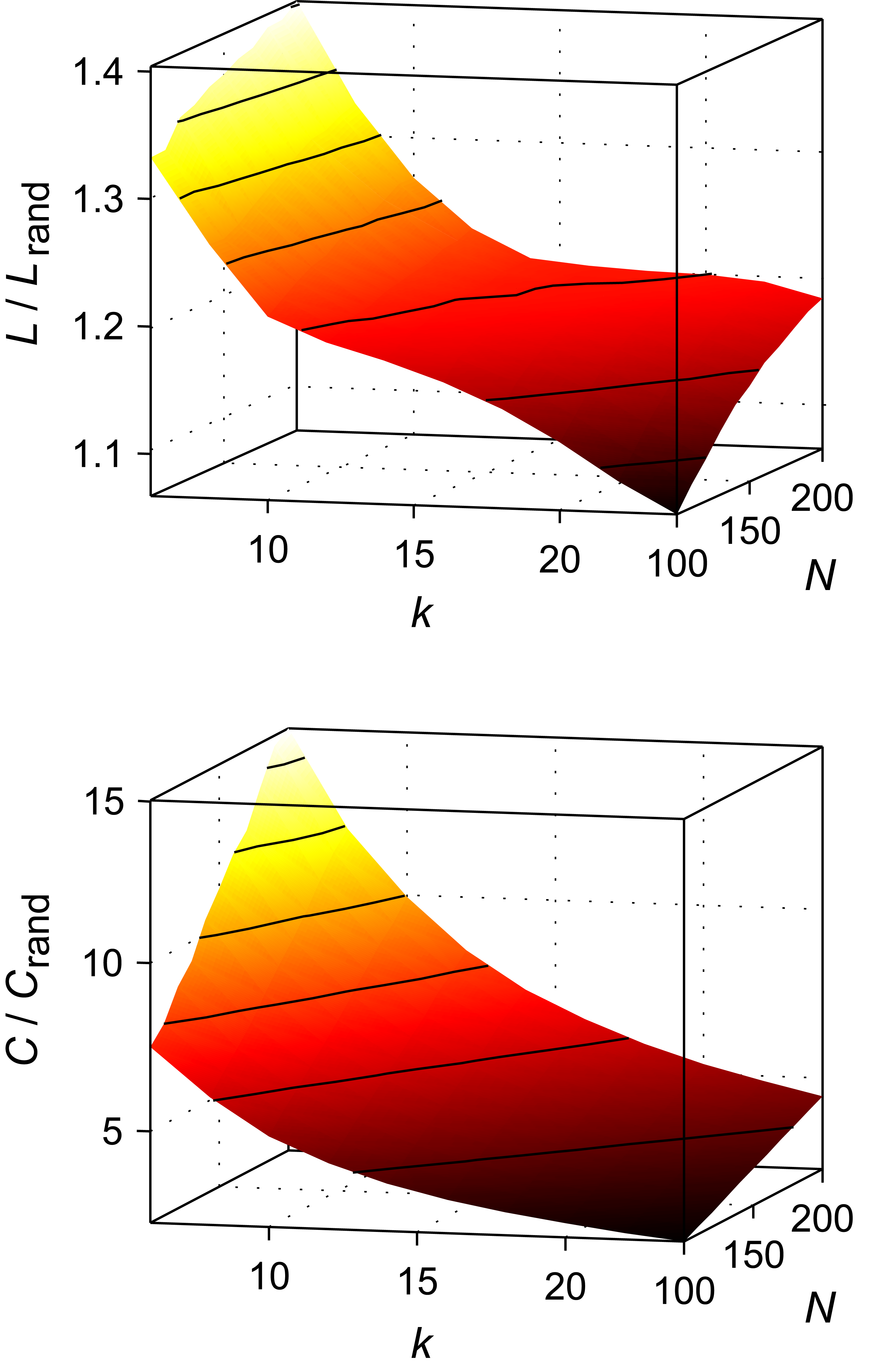

Supplement: Figure S2 — 3D surface plots of the relation between changes in network size and average degree. Increasing only the number of nodes (N) or average degree (k) introduces a change in (normalized) path length (L) and clustering coefficient (C). Same values can only be reached by adjusting both the number of nodes and average degree at the same time. Shown here for a small-world network with a rewiring probability of 0.1. Contour lines are plotted on top for better visualization. (3.76 MB TIF) [file pone.0013701.s003.tif]

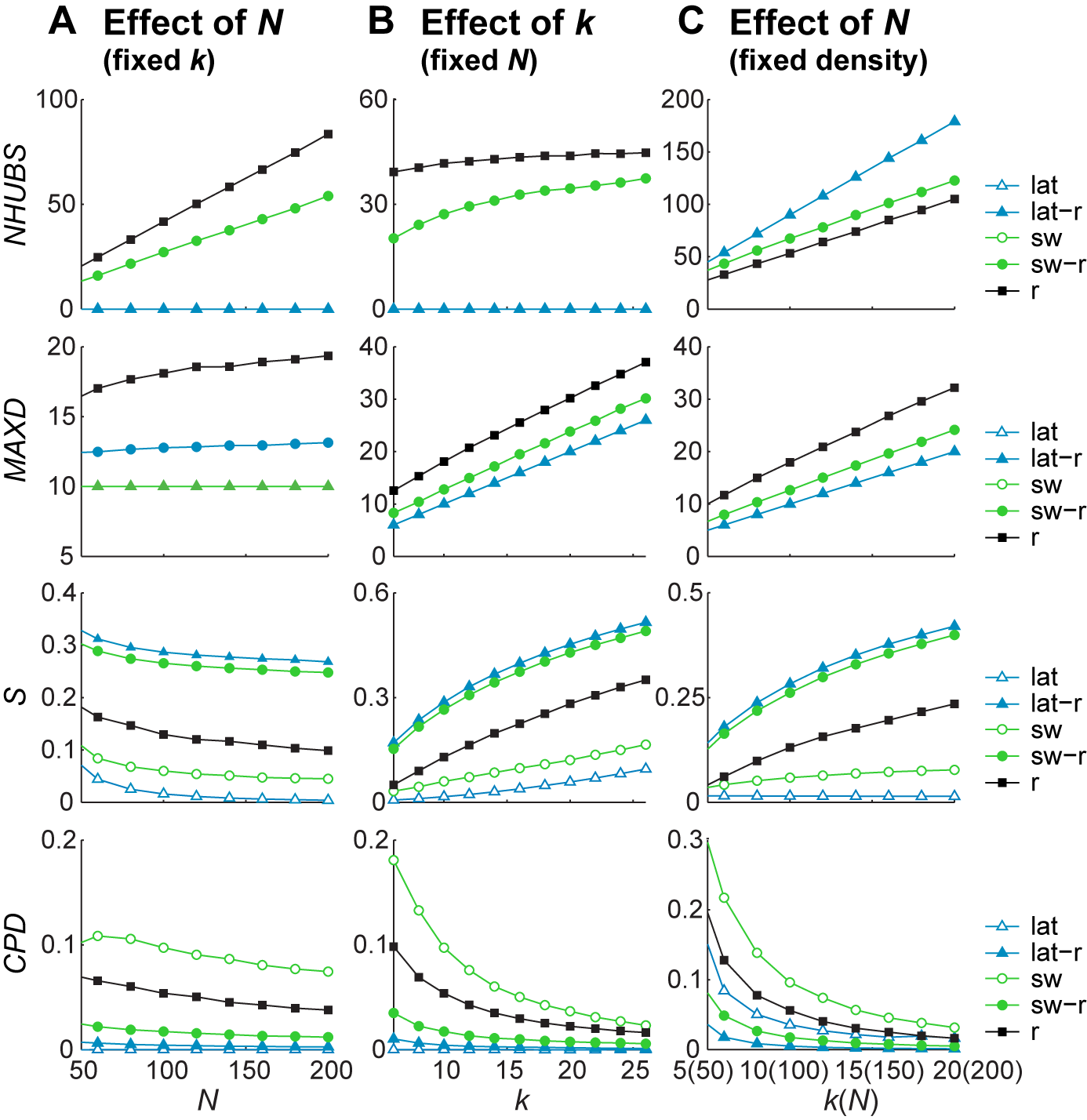

Supplement: Figure S3 — Sensitivity of other graph measures to changes in network size and average degree. The path length, clustering coefficient and small-world index in the main text's Figure 3 are not the only graph measures showing N,k-dependencies that are specific to the type of network. The number of hubs (NHUBS) scales linearly with the number of nodes in a network. The occurrence of hubs in lattices here for the right plot results from the fact that the average degree is adapted for each N to preserve the fixed edge density. The k-values on the x-axis are rounded, the real values are non-integers leaving some nodes to have one edge extra than others and as a consequence are classified as ‘hubs’. The maximum degree (MAXD) naturally increases with the number of edges in the network. Synchronizability (S) and central point dominance (CPD) mainly depend on the network's average degree, so that a fixed wiring cost cannot reduce the independence from changes in network size. For all measures with N,k-dependencies that are specific to the type of network, normalization by random graphs will by construction lead again to N,k-dependent measures. Networks here either have a fixed average degree k = 10, fixed number of nodes N = 100 or a fixed edge density = 0.1. lat, lattice; lat-r random network with uniform degree distribution; sw, small-world network with a rewiring probability of 0.1; sw-r, random network with same degree distribution as sw; r, Erdös-Rényi random network. Exact definitions of all measures can be found in Text S1. (5.98 MB TIF) [file pone.0013701.s004.tif]

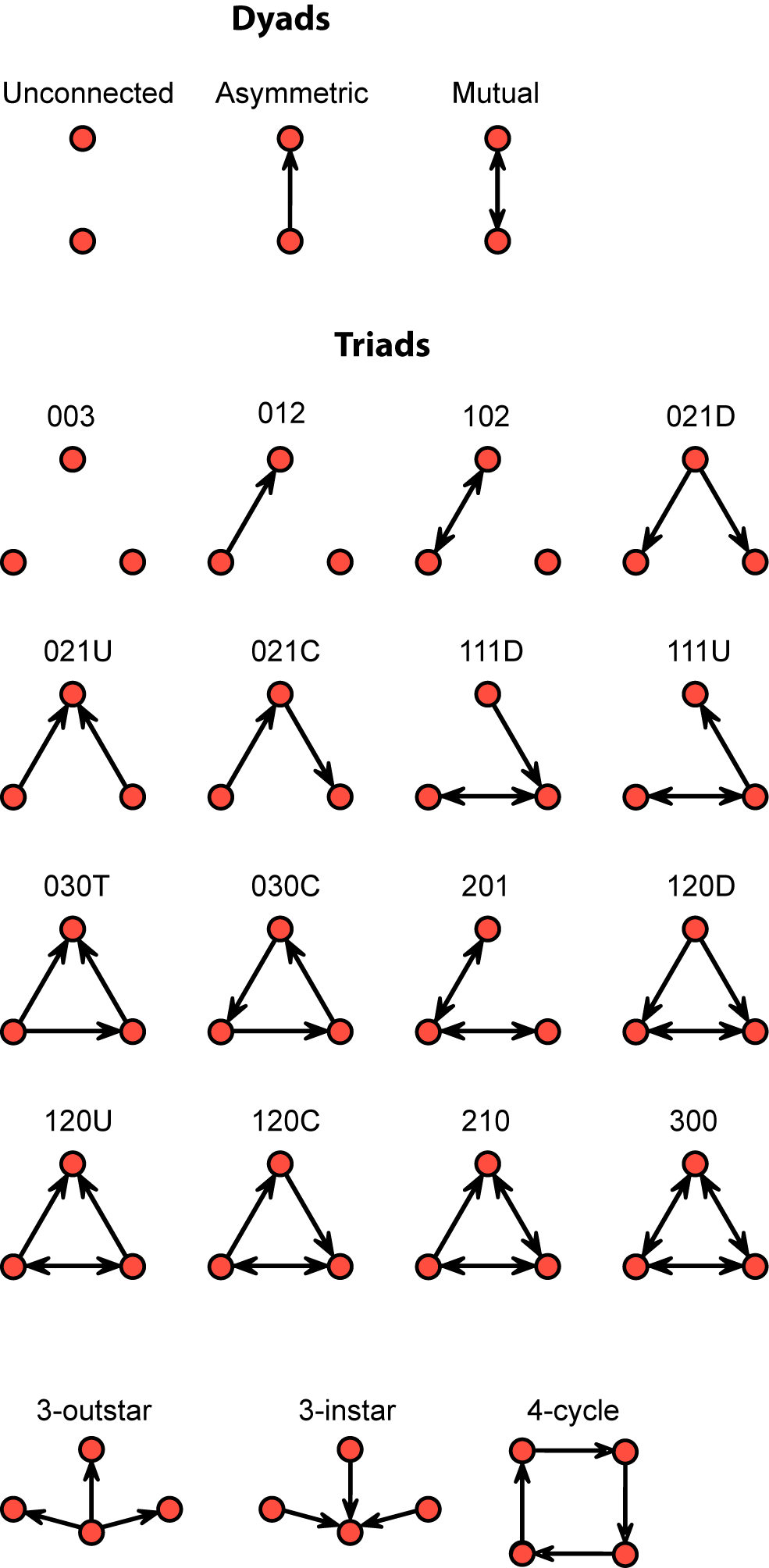

Supplement: Figure S4 — Examples of directed network motifs. Shown are all possible dyads, triads and examples of k-instars, k-outstars and k-cycles. The number of possibilities rapidly increases for motifs with more than 3 nodes. These and other motifs could in principle all be used for both exponential random graph models and motif counting. (5.96 MB TIF) [file pone.0013701.s005.tif]

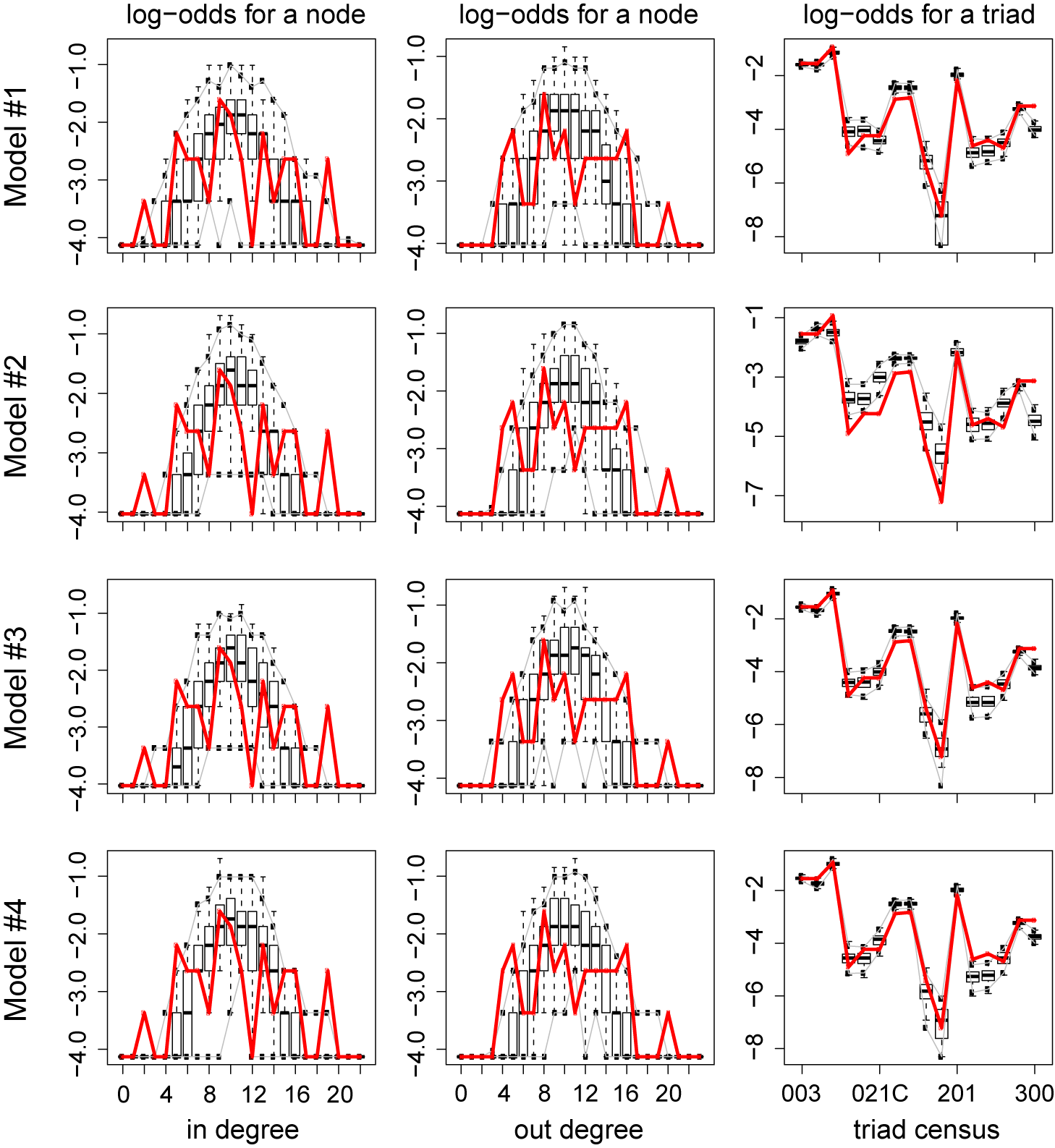

Supplement: Figure S5 — Goodness-of-fit diagnostics for the four exponential random graph models. The red lines represent the statistics of the observed network. The distribution of 100 networks simulated with the estimated parameter values of the model is indicated by the boxplots. The grey lines represent 95% confidence intervals. For the order of the triad census, see Figure S4. (6.99 MB TIF) [file pone.0013701.s006.tif]
